# Evaluation of variable new antigen receptors (vNARs) as a novel cathepsin S (CTSS) targeting strategy

**DOI:** 10.3389/fphar.2023.1296567

**Published:** 2023-12-05

**Authors:** P. Smyth, L. Ferguson, J. F. Burrows, R. E. Burden, S. R. Tracey, Ú. M. Herron, M. Kovaleva, R. Williams, A. J. Porter, D. B. Longley, C. J. Barelle, C. J. Scott

**Affiliations:** ^1^ Patrick G. Johnston Centre for Cancer Research, Queen’s University Belfast, Belfast, United Kingdom; ^2^ Elasmogen Ltd., Aberdeen, United Kingdom; ^3^ School of Pharmacy, Queen’s University Belfast, Belfast, United Kingdom; ^4^ Scottish Biologics Facility, Institute of Medical Sciences, School of Medicine, Medical Sciences and Nutrition, University of Aberdeen, Aberdeen, United Kingdom

**Keywords:** therapeutic, cancer, inhibitor, vNAR, cathepsin

## Abstract

Aberrant activity of the cysteine protease Cathepsin S (CTSS) has been implicated across a wide range of pathologies. Notably in cancer, CTSS has been shown to promote tumour progression, primarily through facilitating invasion and migration of tumour cells and augmenting angiogenesis. Whilst an attractive therapeutic target, more efficacious CTSS inhibitors are required. Here, we investigated the potential application of Variable New Antigen Receptors (vNARs) as a novel inhibitory strategy. A panel of potential vNAR binders were identified following a phage display panning process against human recombinant proCTSS. These were subsequently expressed, purified and binding affinity confirmed by ELISA and SPR based approaches. Selected lead clones were taken forward and were shown to inhibit CTSS activity in recombinant enzyme activity assays. Further assessment demonstrated that our lead clones functioned by a novel inhibitory mechanism, by preventing the activation of proCTSS to the mature enzyme. Moreover, using an intrabody approach, we exhibited the ability to express these clones intracellularly and inhibit CTSS activity whilst lead clones were also noted to impede cell invasion in a tumour cell invasion assay. Collectively, these findings illustrate a novel mechanistic approach for inhibiting CTSS activity, with anti-CTSS vNAR clones possessing therapeutic potential in combating deleterious CTSS activity. Furthermore, this study exemplifies the potential of vNARs in targeting intracellular proteins, opening a range of previously “undruggable” targets for biologic-based therapy.

## Introduction

Amongst lysosomal cysteine cathepsins, cathepsin S (CTSS) holds particular interest due to a range of distinctive properties including a normal restricted expression profile, inducible upregulation and activity across a broad pH range (Kirschke et al., 1989; Hsing and Rudensky, 2005; Turk et al., 2012; Wilkinson et al., 2015; Kramer et al., 2017). CTSS has been shown to play an important role in a number of biological processes, most notably in the degradation of redundant proteins and, within immune cells, where it facilitates MHC-II processing through CD74 cleavage (Riese et al., 1996; Nakagawa et al., 1999; Hsing and Rudensky, 2005; Smyth et al., 2022). When not tightly controlled however, aberrant CTSS expression and activity have been demonstrated in a variety of pathologies, ranging from cardiovascular disease to respiratory conditions and cancer, marking it out as both a biomarker and potential therapeutic target (Smyth et al., 2022). In cancer CTSS has been found to be of importance across a range of malignancies including in colorectal, breast and pancreatic cancers. It has been demonstrated to accelerate tumour progression through angiogenesis, where it has a collagenolytic and elastolytic function and by contributing to tumour cell invasion and migration through the degradation of extracellular matrix (ECM), in addition to promoting an inflammatory and in turn, pro-tumourigenic environment (Fernández et al., 2001; Flannery et al., 2003; Shi et al., 2003; Entschladen et al., 2004; Gocheva et al., 2006; Wang et al., 2006; Burden et al., 2009). Of particular note is the ability of CTSS, in contrast to other cysteine cathepsins, to retain functionality at neutral or mildly alkaline pH, facilitating these extracellular proteolytic activities (Vasiljeva et al., 2005; Wartenberg et al., 2019; Smyth et al., 2022).

To elucidate and more accurately understand CTSS mediated effects and roles in disease states, novel and more selective inhibitory molecules are required. In addition, such inhibitors may have clinical applications across the gamut of pathologies in which CTSS is active. Inhibitors should not only possess exquisite target specificity but also retain functionality at lysosomal pH. To date, efforts have overwhelmingly focussed on the development of small molecule inhibitors borne out of either the modification of fluorescent substrate-based activity probes or through the optimisation of inhibitor scaffolds possessing poor selectivity (Gauthier et al., 2007; Drag and Salvesen, 2010; Hilpert et al., 2013; Kasperkiewicz et al., 2014; Poreba et al., 2014). Previously, biologic approaches have also yielded some promising initial findings as demonstrated with the use of the monoclonal antibody Fsn0503 (Burden et al., 2009; Ward et al., 2010) and studies utilising recombinant CTSS propeptide domains (Burden et al., 2008).

One avenue yet to be explored is the application of intrabodies, antibody or antibody fragments which are designed to be expressed intracellularly before being directed to the subcellular location of the target antigen (Lobato and Rabbitts, 2003; Tanaka et al., 2003; Tanaka and Rabbitts, 2003; Stocks, 2005; Marschall et al., 2015; Marschall and Dübel, 2016). To date, the intrabody approach has been used successfully against a range of attractive oncologic targets including epidermal growth factor receptor (EGFR) and vascular endothelial growth factor receptor 2 (VEGF-R2) (Böldicke et al., 2005; Lo et al., 2008; Marschall and Dübel, 2016). The most frequently employed format for intrabodies are single-chain antibodies (scFv fragments) due to relative ease of expression and intracellular stability when compared to full IgG antibodies (Lo et al., 2008; Marschall et al., 2015). Despite this, scFvs are too labile to survive the harsh pH and proteolytic activity found within the lysosome and as such, a more robust biological scaffold is required. One promising alternative are Variable New Antigen Receptors (vNARs).

vNARs, a cornerstone of the adaptative immune system in sharks, are the smallest naturally occurring single chain binding domains in vertebrates and possess distinct properties which make them attractive candidates as biologic inhibitors (Greenberg et al., 1995; Barelle et al., 2009; Barelle and Porter, 2015). Structurally, despite their small size (∼11 kDa), vNARs possess four distinct binding loops (CDR1, CDR3, HV2 and HV4), enhancing propensity for target interaction (Kovaleva et al., 2014). Additionally, these domains have been shown to present as protruding, extended paratopes, with a predisposition to bind cryptic target epitopes, inaccessible to conventional biologics, yielding both specific and tight binders (Nuttall et al., 2002; 2003; Stanfield et al., 2004; Muller et al., 2012; Barelle and Porter, 2015; Kovaleva et al., 2017). Alongside this, borne out of the harsh environment presented by shark sera, is a remarkable inherent stability, allowing retention of molecular integrity in the face of changes in temperature and pH, the presence of organic solvents and enzymatic activity (Dooley et al., 2003; Dooley and Flajnik, 2005; Flajnik et al., 2011; Griffiths et al., 2013; Tracey et al., 2023). Furthermore, vNARs can be expressed in a cost-effective manner in non-mammalian cells at scale. With these features in mind, we proposed that vNARs may be amenable to targeting CTSS, with resistance to protease degradation and the lysosomal pH coupled with attractive binding capabilities.

Here we describe the development and characterisation of anti-CTSS vNARs. vNARs were initially isolated from a vNAR phage display library screened against the CTSS proenzyme. Promising binders identified by phage ELISA were expressed periplasmically and purified, with binding affinity confirmed by ELISA and SPR. Binding affinity was subsequently illustrated to be retained at lysosomal pH. Lead vNAR clones were shown to inhibit the activity of CTSS in fluorescence-based activity assays in a dose dependent manner. Further investigation indicated interestingly that the vNARs could inhibit the activation of the CTSS proform to the mature enzyme. This was further demonstrated in *in vitro* cell-based assays utilising an intracellular vNAR expression system. This study exemplifies vNARs as novel CTSS binders, with potential utility as both tools for further investigating CTSS biology and as therapeutics.

## Methods

### Antigen preparation

Human recombinant proCTSS was produced by Fusion Antibodies Plc. This protein was designed with an active site mutation (C25S) to prevent autocatalytic processing to the active protease species, thereby providing a stable antigen and is subsequently referred to in the text as proCTSS^C25S^ (Turkenburg et al., 2002). To facilitate panning, proCTSS^C25S^ antigen was biotinylated. Briefly, Sulfo-NHS-LC-Biotin (ThermoFisher) was removed from freezer storage and equilibrated to room temperature. Biotin reagent was subsequently prepared as a 10 mM stock concentration in ultrapure water before adding an appropriate volume to the CTSS antigen. The reaction was then incubated on ice for 2 h before being dialysed overnight in 1x PBS. Success of the biotinylation process was confirmed through a biotinylation-depletion assay.

### Phage displaying panning process

vNAR clones against proCTSS^C25S^ were isolated from Elasmogen’s proprietary synthetic, multiframework vNAR libraries, maintained in Elasmogen’s proprietary phagemid vector. Panning was conducted against biotinylated proCTSS^C25S^ which was captured on M-280 Streptavidin Dynabeads (Thermo Fisher Scientific). Phage display panning was carried out as previously described (Ubah et al., 2021). Antigen concentration was decreased through the panning process to increase stringency, with 400 nM, 200 nM, 50 nM, and 50 nM employed through rounds 1, 2, 3 and 4 respectively. Following the final panning round, individual clones were selected, inoculated into supplemented 2xTY broth and grown overnight. Cultures were subsequently co-infected with M13 helper phage and PEG precipitated, yielding a panel of monoclonal vNAR-presenting phage. These were subsequently screened for binding via a direct phage binding ELISA.

### Phage ELISA

High binding Nunc Maxisorp 96-well ELISA plates were coated with target antigen (100 µL/well) at 1 μg/mL in PBS and incubated overnight at 4°C. Plates were subsequently washed three times using 0.1% PBST and blocked for 1 h at room temperature in 3% MPBS (3% w/v milk (Marvel) in 1x PBS). On completion of plate blocking, wells were again washed three times with 0.1% PBST (0.1% v/v Tween-20 in × 1 PBS) before addition of blocked phage (100 µL/well) for 1 h at room temperature. Plates were subsequently washed as previously described before the addition of anti-M13-HRP antibody (Sino Biological), (diluted 1:4,000 in PBST) for 1 h at room temperature (100 µL/well). After further washing, 100 µL/well TMB substrate solution was added before reaction neutralisation using 50 µL/well 1 M H_2_SO_4_. Absorbance was then read at 450 nm. ELISA was simultaneously conducted on a control plate similarly coated with HSA.

### Periplasmic expression and IMAC purification

vNARs were expressed periplasmically in TG1 cells. An initial starter culture of TG1s (20 mL) was used to inoculate 1 L of Terrific Broth (TB) supplemented with phosphate salt, 1% glucose and 100 μg/mL ampicillin. Cells were incubated for 7 h at 37°C with vigorous shaking, at which point cells were pelleted by centrifugation at 4,000 x *g* for 20 min (20°C). Following centrifugation, the bacterial pellet was resuspended in 1 L of fresh TB supplemented with phosphate salt, 1% glucose and 100 μg/mL ampicillin. This was left to incubate o/n with vigorous shaking at 30°C. Following o/n culture, cells were pelleted by centrifugation as previously described before resuspension in 1 L of fresh TB supplemented with phosphate salt, 1 mM IPTG and 100 μg/mL ampicillin. The culture was then incubated, with shaking, for 4 h at 30°C. Following this incubation step, cells were again pelleted by centrifugation at 6,000 x *g* for 30 min. The cell pellet was then resuspended in 100 mL ice-cold TES buffer (200 mM Tris/HCl, 1 mM EDTA pH8.0, 20% Sucrose pH8.0) before gentle shaking (100 rpm), on ice, for 15 min. After 15 min, an equal volume of ice-cold MgSO_4_ was added to a final concentration of 2.5 mM. The cell suspension was then incubated on ice for a further 15 min. Next, the suspension was pelleted by centrifugation at 6,000 x *g* for 30 min (4°C) and the supernatant containing the released periplasmic extract collected. Expressed vNARs were then purified by Immobilised Metal Affinity Chromatography (IMAC). Briefly, 2 mL of Nickel resin (His Pur Ni-NTA Resin, Thermo Fisher#88222) was added to 200 mL of periplasmic extract and mixed on a roller at room temperature for 2 h. Periplasmic extract was then allowed to pass through a 10 mL chromatography column (Bio-Rad# 731-1550). The trapped resin was subsequently washed, first with 40 mL sterile PBS, before washing with 20 mL 10 mM imidazole. vNAR was finally eluted with 5 mL 500 mM imidazole (pH 8) and the eluate collected. Imidazole was removed from the eluted fraction via dialysis in 2 × 5 L PBS with agitation at 4°C in a dialysis cassette (Slide A Lyzer Dialysis cassette 7,000 MWCO; 0.5–3 mL capacity, Thermo Fisher #66370).

### Periplasmic ELISA

Post-expression, vNAR binding was assessed by ELISA. NUNC Maxisorp 96-well ELISA plates were coated with proCTSS^C25S^ antigen or HSA at 1 μg/mL in PBS (100 μL/well) and incubated o/n at 4°C. ELISA plates were subsequently washed three times with 0.1% PBST (0.1% v/v Tween-20 in 1x PBS), before being blocked with 4% M-PBST (300 μL/well) for 1 h at RT. On the completion of the blocking step, the plate was washed as before (three times with PBST), 100 µL of vNAR (PBS) added per well and incubated for 2 h at RT. The plate was subsequently again washed thrice with PBST before 100 μL/well anti-Myc-Peroxidase (Roche, 11814150001) was added and incubated at RT for 1 h. Plates were next washed three times with PBST and three times with PBS prior to the addition of 100 μL/well TMB substrate. Upon colorimetric change, the reaction was halted via the addition of 50 μL/well 1 M H_2_SO_4_. Absorbance was then read at 450 nm using a microplate absorbance reader.

### Binding affinity determination

For SPR binding assessment, experiments were conducted at 25°C on a Biacore 8K instrument. HBS-EP + running buffer (Cytiva) was used unless otherwise stated. Initial carboxymethylated dextran CM5 sensor chip (Cytiva) activation was achieved following the addition of 0.4 M EDC and 0.1 M NHS. The activated chip was subsequently functionalised with 20 μg/mL proCTSS^C25S^ protein prepared in 10 mM sodium acetate buffer (pH 5). Following this, remaining NHS esters were quenched through the addition of 1 M ethanolamine hydrochloride (pH 8.5). Chip activation, functionalisation and quenching steps were each conducted with a constant flow rate. vNAR samples were diluted as required in HBS-EP running buffer and flowed over the immobilised antigen at 30 μL/min for 30 s. Bound vNAR was then allowed to dissociate for 300 s. The sensor chip was regenerated between sample injections by stripping bound vNAR via three sequential sodium hydroxide treatments (50 mM at 30 μL/min for 30 s).

### Recombinant CTSS enzyme assays

For all recombinant CTSS enzyme assays, CTSS (R&D, 1183-CY) was utilised. Fluorometric activity assays were conducted in triplicate in black 96-well microtitre plates. Activity assay buffer was comprised of 100 mM sodium acetate, 1 mM EDTA, and 5 mM DTT (pH 5.5) for activation of CTSS (R&D, 1183-CY). Unless otherwise stated, CTSS was pre-activated in assay buffer for 25 min, with shaking, at 37°C prior to addition to assay. Activity was monitored using the fluorogenic substrate Z-VVR-AMC (BML-P199, Enzo). Purified vNARs were added to assays at various concentrations as detailed. Each well contained 10 ng enzyme and 10 μM substrate, with volumes equilibrated using activity assay buffer. Fluorescence, as a measure of substrate turnover, was monitored over a 1 h period with readings taken at 2 min intervals (ex380/em460). Fluorometric assays utilising 100 μg/mL fluorescent DQ-gelatin (Invitrogen) were conducted as above (ex480/em530).

For western blots, CTSS was activated as described above, in assay buffer comprised of 100 mM sodium acetate, 1 mM EDTA, and 5 mM DTT (pH 5.5). For non-activated samples, CTSS was incubated in assay buffer comprised of 100 mM sodium acetate and 1 mM EDTA (pH 5.5). Enzyme was incubated alongside vNAR clones as detailed in the figure legends.

### Cell-line culture

HCT116, RAJI and U251 cells were acquired from ATCC. RAJI cells were cultured in RPMI-1640 supplemented with 10% FCS and 1% penicillin/streptomycin. U251 cells were maintained in high glucose DMEM supplemented with 10% FCS and 1% penicillin/streptomycin. HCT116 cells were cultured in McCoy’s medium supplemented with 10% FBS, 1% penicillin/streptomycin and 1% sodium pyruvate. All cells were maintained in a humidified environment containing 5% CO_2_ at 37°C.

### vNAR expression vector

pDQ-EV vector was used as previously described. Cloning of CTSS vNAR sequences into plasmids was conducted using standard PCR protocols, with PCR products separated on 2% agarose gels and appropriate bands excised and purified with QIAquick Gel Extraction Kit (Qiagen) and QIAquick PCR Purification Kit (Qiagen) respectively as per manufacturers’ instruction (McFarlane et al., 2010; de la Vega et al., 2011; Jaworski et al., 2014; McCann et al., 2018; Lin et al., 2022). Primers were designed allowing insertion of Kozak, the natural CTSS signal peptide motifs and vNAR sequence as per Elasmogen’s proprietary library. Cell transfections were conducted using Lipofectamine™ 3,000 (Invitrogen) and Amaxa Nucleofector (Lonza) transfection systems as per the manufacturers’ instruction. Cell viability was determined via trypan blue at point of seeding, with viability of at least 90% for all studies. Post-transfection, cell viability was assessed visually. Briefly, Raji cells were transfected via 4D-Nucleofector X Unit. Raji cells at 2 × 10^6^ cells/mL were centrifuged and resuspended in 100 μL of Nucleofector X solution, prior to addition of 5 μg of plasmid DNA. Cell suspension was then added to the Nucleocuvette and transfected using program DS-104. Post transfection, 400 μL of prewarmed culture media was added to the suspension prior to transferring to 1 mL of pre-warmed culture media in a 6-well tissue culture plate. Following transfection, visual assessment confirmed viability of ∼70%. Likewise, with Lipofectamine 3,000 (HCT116), viability post-transfection was again found to be ∼70%. Here, the cell media was replaced 6 h post-transfection to minimize transfection toxicity. Transfection was performed in accordance with the manufacturers’ direction. Cells were seeded in 6-well tissue culture plates at 1 × 10^6^ cells/mL. Lipofectamine reagent was added to DNA (5 μg) in Opti-MEM media, and allowed to incubate prior to addition to cells.

### Western blot analysis

Cells were lysed, collected and quantified via bicinchoninic acid (BCA) assay (Pierce, United Kingdom) as previously described (Leach et al., 2020). Prior to gel electrophoresis, protein samples were denatured at 95°C for 10 min in X5 Laemmli buffer, before separation by SDS-PAGE at 120 V. Total protein concentration was equalised across each well for all Western blot analysis. For cell lysates 60 μg of protein was loaded per well. This was reduced to 0.025 μg for assessment of recombinant CTSS protein. Following the completion of the sample running, a semi-dry transfer (BioRad) onto a polyvinylidene fluoride membrane (Millipore, United Kingdom) was carried out. This membrane was subsequently blocked (5% w/v milk in TBS-Tween) for 1 h, prior to the addition of primary and secondary detection antibodies, which were used as per the manufacturers’ directions. Primary antibodies were used as follows: goat anti-human-CTSS (RnD Systems, AF-1183) (1:2000) and mouse anti-6X-his (Abcam, ab18184) (1:1000) with subsequent incubation with secondary antibodies donkey anti-goat-HRP (Santa Cruz, sc-2056) (1:5,000) and goat anti-mouse-HRP (Bio-Rad, 172-1011) (1:10,000). Western blots were imaged via the addition of a chemiluminescent substrate (Western Lightning ECL Plus) using a G-Box system (SynGene).

### Invasion assays

*In vitro* invasion assay was conducted as previously described, with 8 μm polycarbonate inserts placed in a 24 well plate (Burden et al., 2009; Wilkinson et al., 2016). The upper chamber of each insert was coated with 1 mg/mL Cultrex in serum free media. The plate was then incubated at 37°C for 4 h to enable polymerisation of the Cultrex matrix. Following this, 2.5 × 10^5^ U251 cells were seeded in serum free media into the upper chamber (250 μL). The lower compartment was filled with 750 μL of 48 h U251 conditioned media. For vNAR treatments, equimolar amounts (3.5 μM) were added to both the upper and lower chamber to circumvent gradient effects. Following a 24 h treatment period, non-invaded cells, found within the inside of the upper chamber were removed before invaded cells were fixed using Carnoy’s fixative for 15 min. The membrane was subsequently dried and stained with 50 ng/mL Hoechst for 30 min. Excess stain was then removed by two sequential washes in H_2_O. The membrane was next cut from the insert before being mounted onto a glass slide using VectaMount (Vector, USA) Each treatment condition was conducted in duplicate. Membranes were imaged on a Leica DM5500 microscope operating with LASX software, with 10 images per membrane captured at ×20 magnification. Cells were counted and results presented as the mean number of invaded cells per field of view.

### Statistical analysis

Results presented as mean +/- standard deviation and representative of at least two independent experiments unless otherwise stated. One-way ANOVA was used to assess statistical significance unless otherwise stated. Significance values were calculated using the GraphPad software. *p*-values were considered statistically significant: *< 0.05, **< 0.01.

## Results

To isolate CTSS specific domains from the synthetic vNAR library, a phage display panning approach was employed. Four sequential panning rounds were conducted utilising a liquid phase panning methodology, wherein biotinylated catalytically inactive but stable proCTSS^C25S^ was immobilised on magnetic streptavidin beads before exposure to Elasmogen’s proprietary next-generation synthetic, multi-framework (ELSS) vNAR libraries. Libraries were constructed by fusing naive vNAR frameworks, with sequence diversity introduced within CDR1 and CDR3. Variation was also created through differing CDR3 lengths and the introduction of non-canonical cysteine residues in CDR1 and CDR3 regions. This multiframework approach results in libraries each containing ∼10 billion clones with considerable diversity (Ubah et al., 2021). Prior to commencement of the panning process, a biotin depletion assay was conducted, which confirmed successful biotinylation of the target antigen (Supplementary Figure S1). To increase the stringency of the selection method and eliminate weaker binders, antigen concentration was dropped through the panning rounds, with 400 nM, 200 nM, 50 nM, and 50 nM employed through rounds 1, 2, 3 and 4 respectively. Wash stringency was kept consistent throughout. Progression of the panning process was monitored through both assessment of phage output titres and calculation of the phage infection efficiency.

**FIGURE 1:**
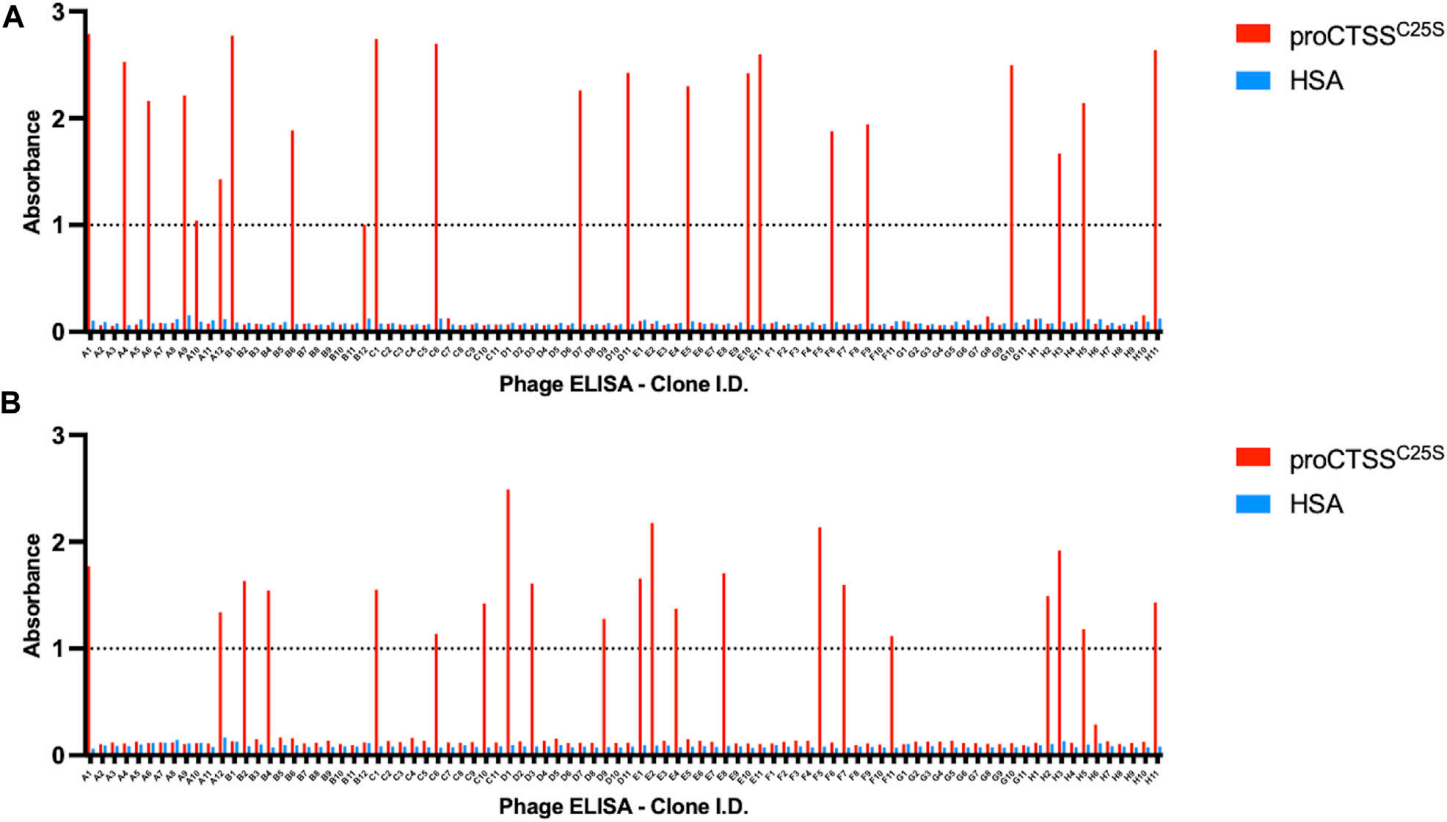
Phage ELISA for proCTSS^C25S^ binding. Following panning, monoclonals were assessed in terms of binding by a phage ELISA. This revealed 44 potential hits across **(A)** ELSS1 and **(B)** ELSS4 libraries, where proCTSS^C25S^ binding was apparent with little non-specific interaction evident toward HSA control wells. A nominal cut-off absorbance value of 1 was employed to narrow down potential hits for further assessment. Data of one phage display ELISA post-panning.

Following the fourth round of panning, 90 individual monoclonals were selected from each library and screened using a phage ELISA against the target antigen. In order to account for non-specific binders, a HSA coated control plate was tandemly assessed (Figure 1).

**FIGURE 2:**
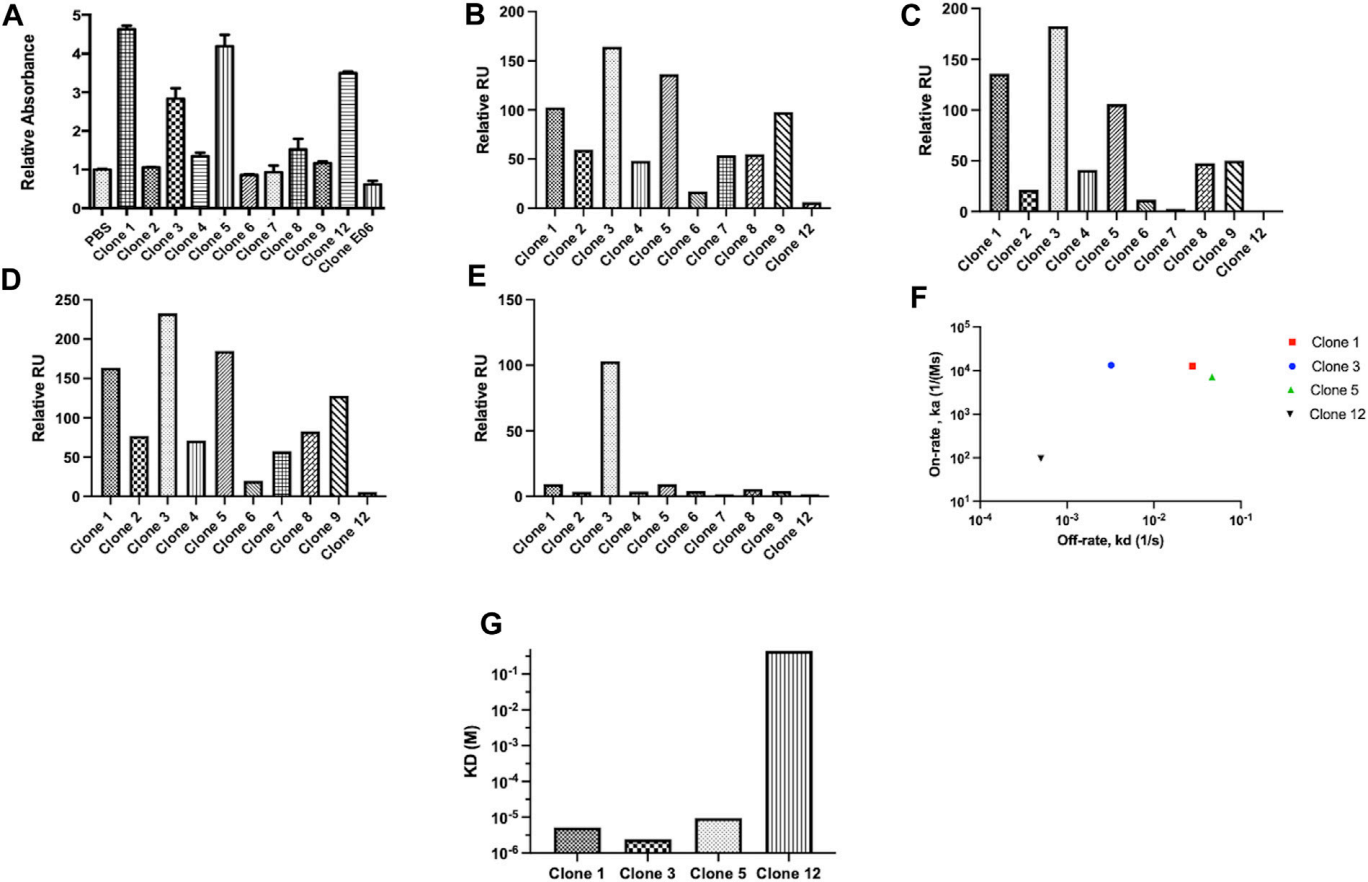
vNAR clone binding assessed following periplasmic expression. Unique clones identified from phage ELISA were taken forward and expressed periplasmically in TG1 cells. Subsequently, clones were subjected to binding assessment via **(A)** ELISA. E06, a HSA specific vNAR clone was included as a control. To confirm binding affinity, proCTSS^C25S^ antigen was immobilised on a carboxymethylated dextran chip and vNAR binding affinity assessed via SPR analysis. Relative binding responses shown for **(B)** analyte binding early (6 s after start of analyte injection), **(C)** analyte binding late (5 s before end of analyte injection), **(D)** analyte stability early (5 s after end of analyte injection) and **(E)** analyte stability late (5 s before end of analyte dissociation period). **(F)** On-rate and off-rate kinetic assessment of lead vNAR clones 1, 3, 5 and 12. **(G)** KD assessment of lead vNAR clones 1, 3, 5 and 12.

From this screen 44 potential binders were identified as determined by having a phage ELISA absorbance value against proCTSS^C25S^ greater than a predetermined relative cut-off of 1. Each of these were individually sequenced, revealing 11 unique clones. These clones were taken forward and expressed periplasmically in TG1 cells. Expression was induced using IPTG and periplasmic proteins were separated from cell debris using osmotic shock. The periplasmic extract was subsequently purified by IMAC purification, exploiting the his-tag present at the C-terminal region of the vNAR clones. The purified products were assessed via Western blot (Supplementary Figure S2). It was found that 10 of the 11 identified clones were successfully expressed with average expression yields of around 4 mg/L. These were then taken forward and screened via ELISA, with binding confirmed using an anti-myc HRP antibody, recognizing the myc tag in the vNAR terminal region (Figure 2A). E06, an HSA specific clone was included as a control for non-specific vNAR binding.

From ELISA screening, differential binding affinities were observed. Notably, clones 1, 3, 5, and 12 exhibited strong affinity for proCTSS^C25S^. To confirm binding affinity, proCTSS^C25S^ antigen was immobilised on a carboxymethylated dextran chip using EDC/NHS coupling chemistry, and vNAR binding affinity assessed via SPR analysis **(** Figures 2B–G; representative sensorgrams—Supplementary Figure S3, lead clone affinity data—Supplementary Figure S4). Briefly, samples were allowed to flow over the immobilised antigen at a flow rate of 30 μL/min for 30 s. Bound analyte was then allowed to dissociate for 300 s. Between samples, the chip was regenerated by stripping bound vNAR via three sequential sodium hydroxide treatments (50 mM at 30 μL/min for 30 s). Encouragingly, binding response trends observed via SPR largely correlated with those seen via ELISA, with lead vNAR clones 1, 3, and 5 again displaying greatest proCTSS^C25S^ binding affinity. Notably clone 12, which displayed high affinity by ELISA, displayed much reduced binding when assessed on the Biacore 8K system, perhaps as a result of proCTSS^C25S^ orientation on the SPR chip surface.

**FIGURE 3:**
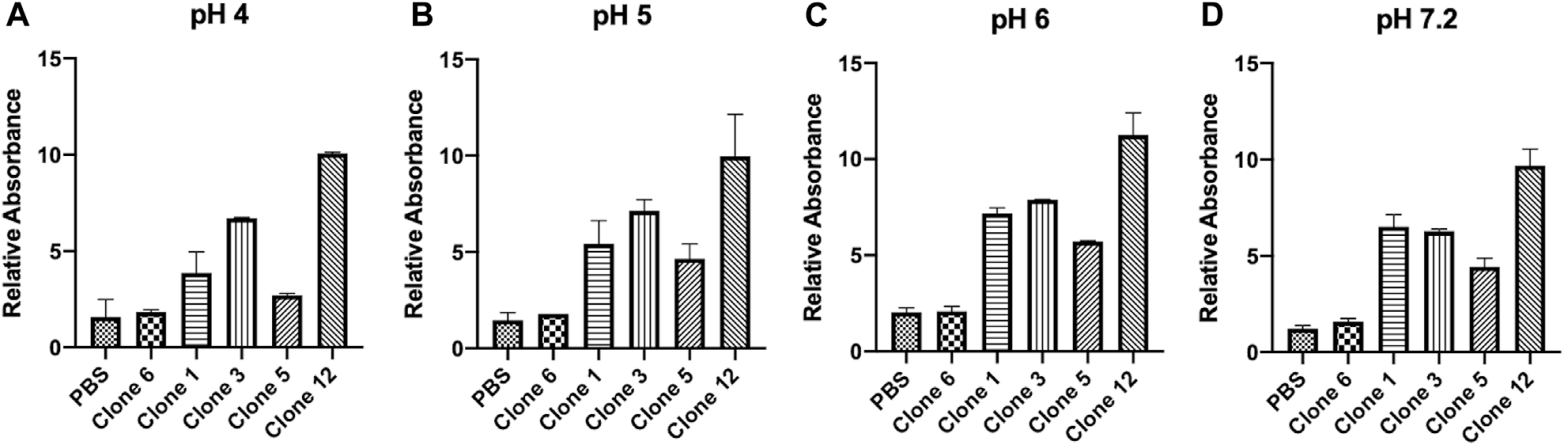
vNARs display stability at acidic pH. vNAR binding to immobilised proCTSS^C25S^ antigen was assessed across a pH gradient via ELISA **(A–D)**. Clone 6, which exhibited minimal binding affinity in previous ELISA and SPR experiments was employed as a negative control. Data representative of three independent experiments.

vNARs have been reported to possess inherent stability against a range of factors including variations in pH, temperature and exposure to proteases (Dooley et al., 2003; Dooley and Flajnik, 2005; Flajnik et al., 2011; Griffiths et al., 2013; Tracey et al., 2023). This stability may be of particular benefit in targeting CTSS, located within acidic lysosomal compartments. To ascertain vNAR stability and the retention of binding affinity across a pH range indicative of the endo/lysosomal pathway, a vNAR binding ELISA was conducted (Figure 3). Clone 6, which exhibited minimal binding affinity in previous ELISA and SPR experiments was employed as a negative control. Promisingly, all lead vNAR clones exhibited minimal reduction in binding affinity upon reduction in pH, even under the harshest of conditions assessed (pH 4).

**FIGURE 4:**
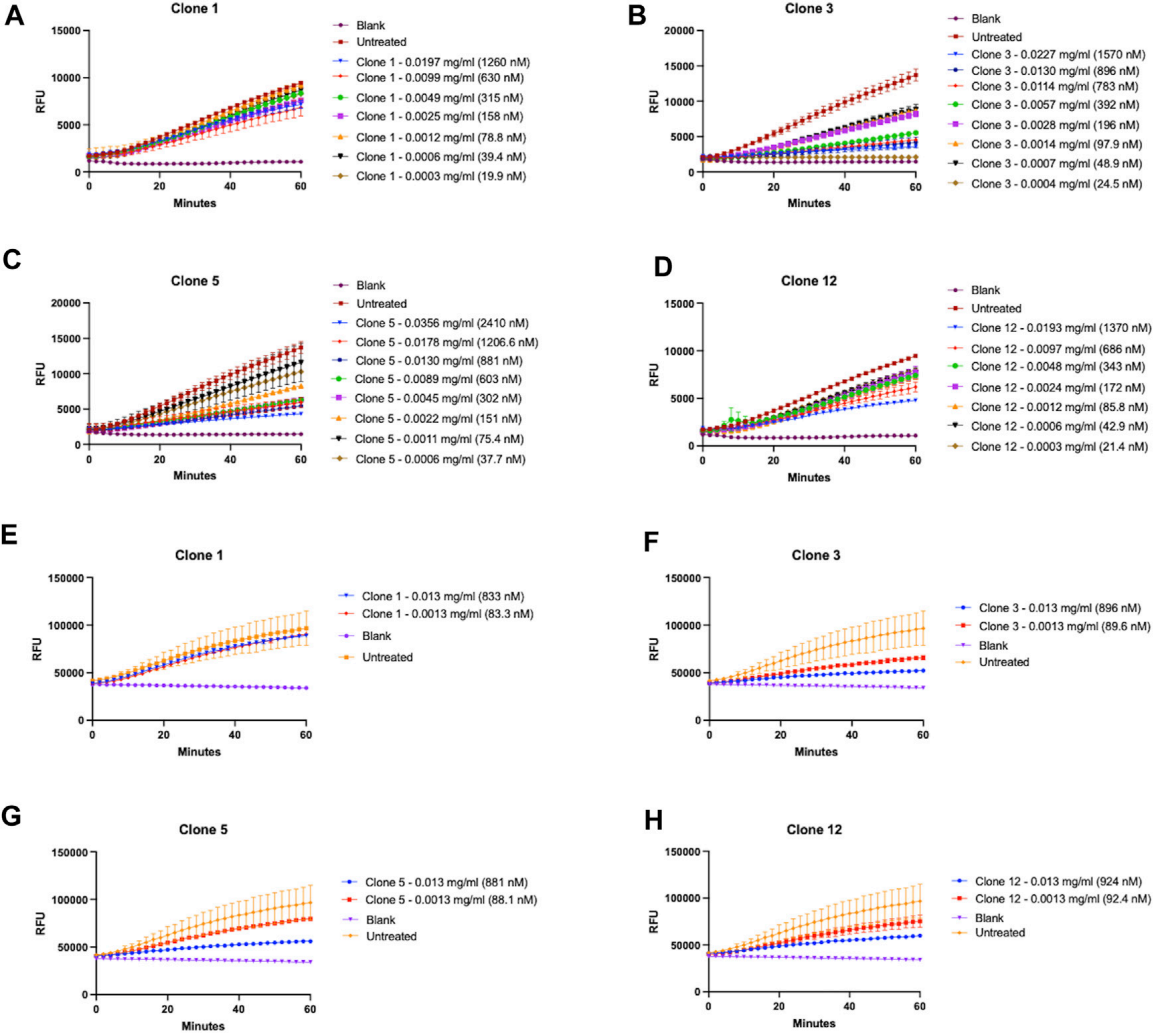
Lead vNAR clones inhibit CTSS enzymatic activity. vNAR clone mediated inhibition of activated recombinant CTSS was first assessed via fluorescence-based activity assays (pH 5.5) using **(A–D)** Z-VVR-amino methyl coumarin and **(E–H)** DQ-gelatin as substrates respectively. CTSS was pre-activated by incubation at 37°C, in assay buffer for 25 min. Substrate turnover was monitored across a 1 h incubation (37°C) with fluorescence measured at 2 min intervals. Relevant control groups are as indicated (blank—assay buffer and substrate in absence of vNAR and CTSS; untreated—assay buffer, substrate and CTSS in absence of vNAR). Data representative of three independent experiments.

Whilst we had successfully demonstrated that our vNARs were capable of binding the proCTSS^C25S^antigen, it was next critical to determine if they were also capable of inhibiting CTSS activity. This functionality was first assessed via a fluorescence-based activity assay employing an internally quenched peptide substrate, Z-VVR-amino methyl coumarin and activated recombinant CTSS. Activity was assessed over a 1 h period with fluorescence measured at 2 min intervals at 37°C. CTSS enzyme was pre-activated by incubation at 37°C, in assay buffer for 25 min prior to commencement of the assay. Importantly, the activity assay was conducted at pH 5.5, making it more representative of lysosomal conditions. This assay revealed that lead clones from the preceding binding assays, namely, clones 1, 3, 5, and 12, exhibited the most apparent inhibition of substrate turnover, with this inhibition shown to be vNAR dose dependent (Figures 4A–D). In particular, clones 3 and 5 proved particularly effective with IC50 values of 344.9 nM and 183.0 nM respectively.

Encouragingly, the fact that not all clones showed inhibition and the differences in responses between the clones indicated that the inhibitory effect observed was as a result of specific binding interaction between vNAR and the epitope on the target, and not merely just a non-specific vNAR effect (Supplementary Figure S5).

As a further assessment of inhibition of CTSS activity, clones were assessed again using DQ-gelatin, a more physiologically relevant substrate (Figures 4E–H). CTSS has been shown to possess gelatinolytic and collagenolytic activity, as evidenced in the tumour microenvironment wherein it contributes to the degradation of extracellular matrix and subsequent cell migration and invasion (Shi et al., 2003; Wang et al., 2006). Lead vNAR clones were shown capable of inhibiting recombinant CTSS mediated degradation of DQ-gelatin. This was particularly noticeable in the presence of clones 3 and 5.

**FIGURE 5:**
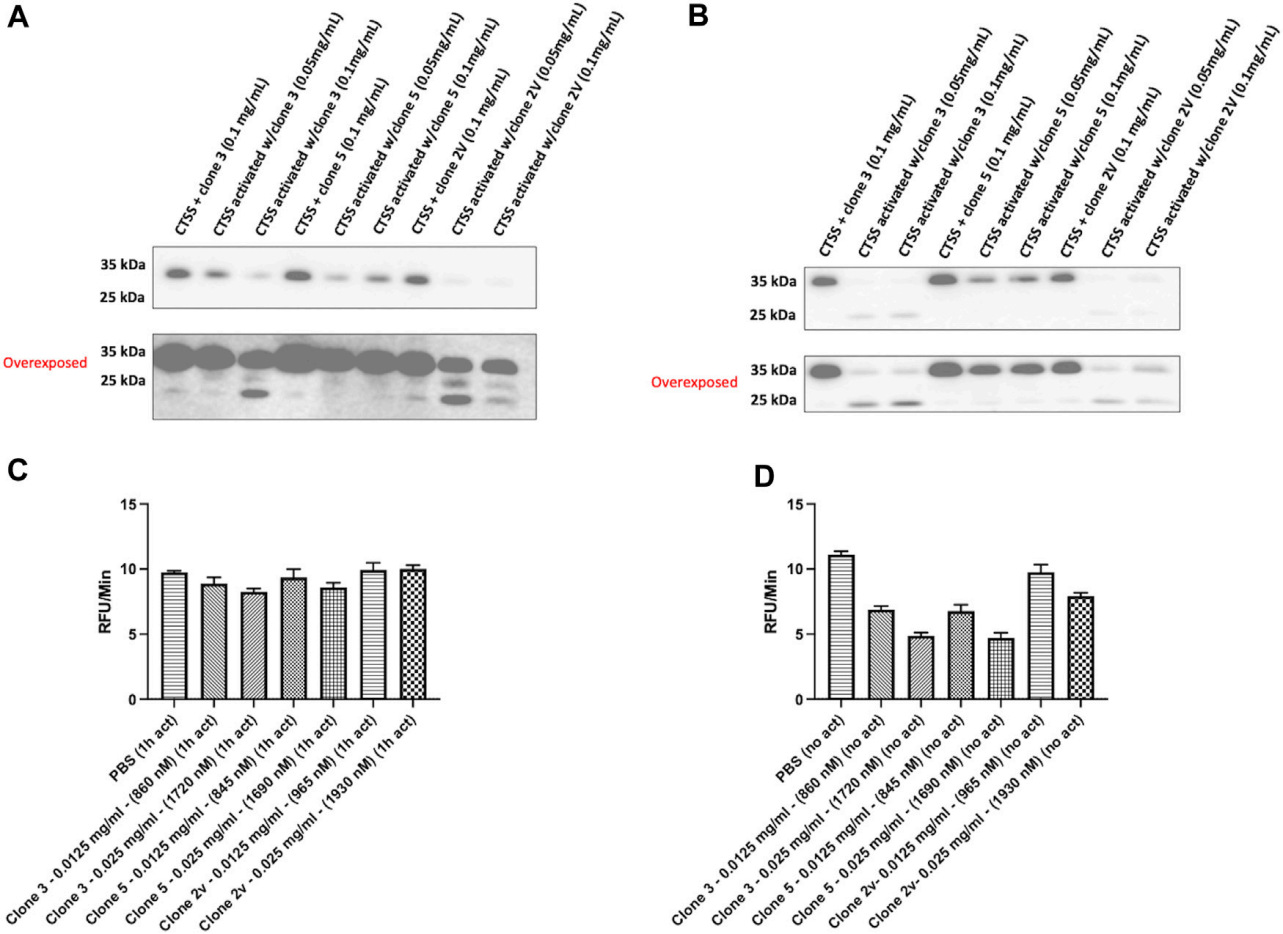
Lead vNAR clones impede CTSS activation from zymogen proform to active species. CTSS (25 ng) was activated alongside anti-CTSS vNAR clones 3 or 5, or isotype control vNAR, 2 V, for either **(A)** 1 h or **(B)** 2 h and CTSS activation status assessed via Western blot. Non-activated CTSS enzyme was included as a control. Z-VVR-amino methyl coumarin turnover by CTSS (R&D Systems) was assessed over a 2 h period, with CTSS enzyme pre-activated for **(C)** 1 h prior or **(D)** no prior activation. Data presented as rate of substrate turnover (RFU/min). Data representative of three independent experiments.

We next sought to repeat our binding assessment against activated CTSS. Interestingly however, binding was not detected by any method (data not shown) even with our highest affinity clones 3 and 5. This led us to hypothesise that, as a result of panning against proCTSS^C25S^, our vNAR binding and inhibitory activity was as a result of a specific interaction with the CTSS proform. To understand this, CTSS was activated in the presence of lead anti-CTSS vNAR clones 3 or 5, or isotype control vNAR, 2V, for either 1 h or 2 h. 2 V is a random naïve domain, originating from naïve spiny dogfish spleen tissue during vNAR library construction (Muller et al., 2012; Smith et al., 2012). Non-activated CTSS enzyme was included as a control. Western blot analysis revealed that anti-CTSS vNARs impeded activation of proCTSS (∼37 kDa) to the mature enzyme (∼25 kDa). Similar effects were not observed with the 2 V control vNAR, where instead, protease activation proceeded unimpaired (Figures 5A, B), in a manner similar to CTSS alone (Supplementary Figure S6). Importantly, under these conditions, CTSS did not degrade the anti-CTSS vNARs (Supplementary Figure S7). This phenomenon was subsequently assessed via the previously described fluorescence-based activity assay, again employing an internally quenched peptide substrate, Z-VVR-amino methyl coumarin and CTSS. Activity was assessed over a 2 h period with fluorescence measured at 2 min intervals at 37°C. CTSS enzyme was either pre-activated by incubation at 37°C, in assay buffer for 1 h prior to commencement of the assay (increased from the 25 min pre-activation step in Figure 4 to guarantee more activated enzyme) or was not subjected to prior activation, in turn ensuring a greater proportion of proCTSS (Figures 5C, D). Inhibition of activity, was shown to be more pronounced in the absence of prior activation, again indicating predominant anti-CTSS vNAR interaction with the proenzyme.

**FIGURE 6:**
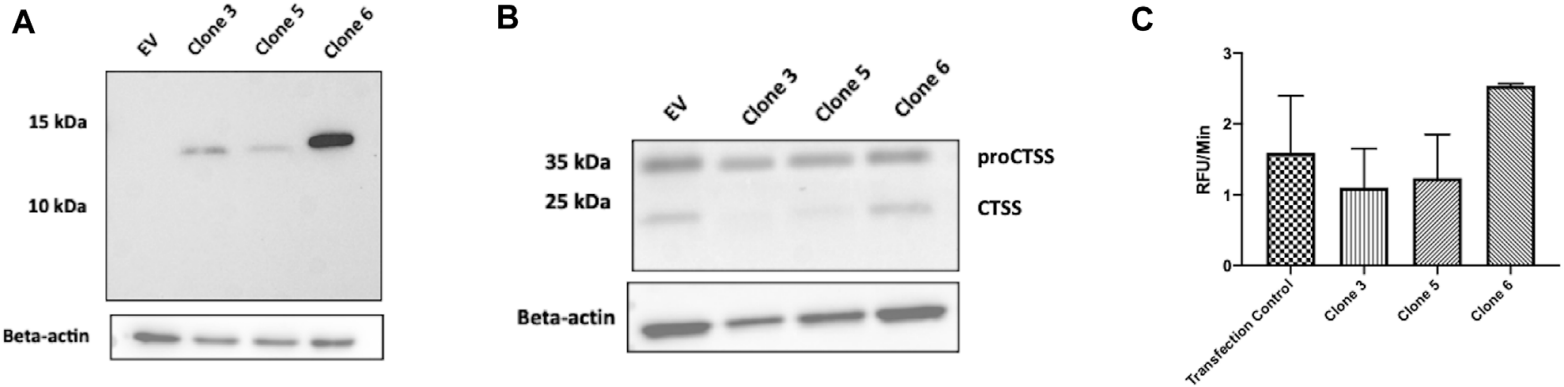
Intracellular expression vectors enable successful translation and function of anti-CTSS vNARs *in vitro*. **(A)** Intracellular expression of vNAR clones 3, 5 and 6 was demonstrated across a panel of cell lines via Western blot (HeLa line shown). **(B)** CTSS Western blot following transfection of vNAR expression vectors and EV (empty vector) control in Raji cells. **(C)** Cell lysates were harvested from Raji cells transfected with vNAR expression vectors or transfection vector alone as control. CTSS from transfected cell lysates were incubated with an internally quenched peptide substrate, Z-VVR-amino methyl coumarin and activity assessed over a 1 h period, with fluorescence measured at 2 min intervals at 37°C.

As an efficient means of delivering our clones intracellularly, the use of an intracellular expression vector was explored. Briefly, vNAR clone sequences were inserted into a pDQ plasmid following the natural CTSS signal peptide motif, to facilitate ER/Golgi trafficking. Following vector design and production, intracellular expression of vNAR clones 3, 5, and 6 following transfection was subsequently demonstrated via Western blot. Following confirmation of expression (Figure 6A), anti-CTSS vNAR mediated inhibition of CTSS activation following transfection of expression vector was demonstrated in Raji cells (Figure 6B). Here, intracellular expression of Clone 3 and 5 vNARs attenuated the activation of the CTSS proenzyme whilst empty vector (EV) and Clone 6, which exhibited minimal binding affinity in previous assays, were employed as negative controls and conversely, did not impede the activation of CTSS.

As further confirmation of the functionality of the vNAR intracellular expression vector, cell lysates were harvested from transfected Raji cells. CTSS from transfected cell lysates was then incubated with Z-VVR-amino methyl coumarin and activity assessed over a 1 h period, with fluorescence measured at 2 min intervals at 37°C (Figure 6C). Lysates from cells transfected with clone 3 and 5 expression vectors displayed reduced CTSS activity when compared to controls (transfection agent alone—Transfection control and clone 6), again indicating inhibition of CTSS activity with our lead molecules.

**FIGURE 7:**
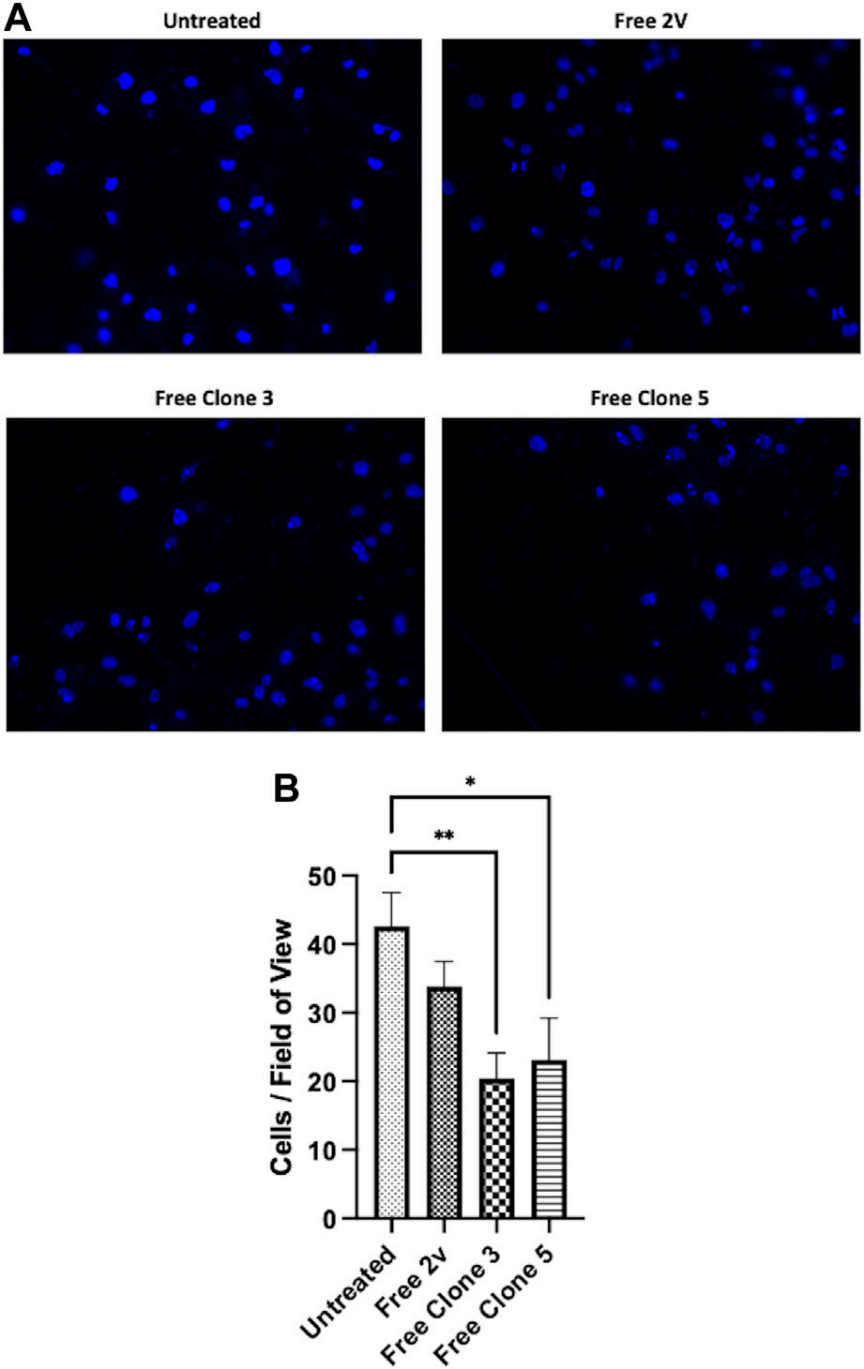
anti-CTSS vNARs reduce extracellular CTSS proteolytic activity. Invasion assay of U251 cells treated with vNARs (3.5 μM) for 24 h. Tumour conditioned media was used to promote cell invasion. Images were captured using a Leica DM5500 microscope operating with LASX software at ×20 magnification. Cell count was performed in 20 fields of view/sample. Findings presented as **(A)** representative invasion images and **(B)** invaded cell counts across 20 fields of view. Data representative of three independent experiments. *< 0.05, **< 0.01.

To exemplify the biological significance of vNAR mediated CTSS inhibition, anti-CTSS vNAR ability to impede cell invasion was assessed using U251 cells (Figure 7). Cell invasion was promoted using tumour conditioned media (TCM) from the respective cells following 48 h in culture. Cells were seeded in serum-free medium and allowed to invade through a Cultrex layer, that mimics the ECM, over a period of 24 h. Cells treated with clone 3 and 5 vNAR protein exhibited reduced invasion when compared to untreated and isotype control 2 V vNAR treated controls highlighting the functionality of the vNARs to attenuate the pro-tumorigenic invasiveness of the cells.

## Discussion

In this study, we have detailed the development and characterisation of a panel of novel vNARs targeted against proCTSS using a variety of activity, biochemical and cellular assays. With potential utility in a range of disease states, the development of specific pharmacological inhibitors of CTSS is an attractive therapeutic strategy. Additionally, unlike other cathepsin family members, the restricted tissue expression (lymphatic tissues and immune cells including professional antigen-presenting cells (APCs) and macrophages) of CTSS makes it a more attractive drug target, as the risk of interfering with regular biological function can be minimised (Kirschke et al., 1989; Hsing and Rudensky, 2005; Turk et al., 2012; Kramer et al., 2017). To date, most of the focus in developing CTSS inhibitors has been on the production of traditional small molecule inhibitors. This has predominantly been explored through the redevelopment and optimisation of subpar inhibitor scaffolds which either lack selectivity or, possess selectivity for another related protease (Gauthier et al., 2007; Hilpert et al., 2013). Alternatively, inhibitors have been produced through the conversion of CTSS activity-based probes via the incorporation of a reactive chemical warhead (James et al., 2003; Drag and Salvesen, 2010; Kasperkiewicz et al., 2014; Poreba et al., 2014). In the development of these inhibitors, emphasis is placed on optimising the P2 position, which has been shown to confer selectivity over other cathepsin family members and similar proteases with the deeper, more flexible S2 pocket in CTSS (as a consequence of Phe211, Gly137 and Val162 residues) providing an attractive target in generating CTSS specific compounds (Pauly et al., 2003; Fuchs et al., 2020; Schade et al., 2020). This approach was used in the design of the Hoffman La Roche compound, RO5459072, which has been investigated in the clinic in inflammatory and autoimmune diseases, such as Sjogren’s syndrome (Sanchez et al., 2010; Hilpert et al., 2013; Hargreaves et al., 2019; Smyth et al., 2022).

Whilst biologic based approaches to target CTSS have been substantially less investigated, encouraging findings have been generated, as exemplified by the monoclonal antibody Fsn0503, which was capable of attenuating invasion and angiogenesis within colorectal tumour models (Burden et al., 2009; Ward et al., 2010). A limitation of such molecules however, is that targeting is limited to extracellular CTSS due to the instability of conventional biologics within lysosomal compartments. As such, these molecules are also too labile to be used in an intrabody based approach and thus we sought to utilise vNARs, due to their well reported inherent stability. To date vNAR application as therapeutics has focussed upon extracellular targets, with vNARs isolated against proteins ranging from ICOSL for use in uveitis, TNF-α in polyarthritis, and AMA1 in malaria, to a variety of potential tumour targets including Her2, DLL4 and PD-1 (Kovaleva et al., 2017; Ubah et al., 2017; 2019; Feng et al., 2019; English et al., 2020; Leach et al., 2020).

More recently, vNARs have been explored for their potential utility in combating the SARS-CoV—2 virus, with clones isolated from a phage display panning process capable of neutralizing pseudotype and authentic live SARS-CoV—2, indicating future therapeutic applications against a range of coronavirus infections (Ubah et al., 2021). Further steps are also underway to optimise vNAR platforms through the application of humanisation strategies and through multimer delivery formats. Moreover, there is also great interest in developing drug delivery systems utilising vNARs as targeting moieties such as targeted nanoparticles and ADCs (Ubah et al., 2018; Cotton et al., 2019; Nogueira et al., 2019; Leach et al., 2020).

Here, we exploited the inherent features of vNARs previously discussed, generating binders against proCTSS^C25S^ through a phage display panning process. Not only did our lead clones show binding to proCTSS^C25S^, via ELISA and SPR based assays, but in established recombinant enzyme activity screens, we observed dose dependent inhibition of CTSS function. Importantly, this was demonstrated not only using an internally quenched peptide substrate, Z-VVR-amino methyl coumarin but also DQ-gelatin, a more physiologically relevant substrate which mimics more closely the ECM. Further, these assays were conducted at lysosomal pH, demonstrating that vNAR clones retained structurally integrity under these conditions, supporting the rationale for their use against intracellular targets.

Further investigation of our lead molecules indicated preferential inhibitory function against the CTSS proform and intriguingly, that our CTSS vNARs were capable of preventing conversion of the proform to the mature enzyme. CTSS exists initially as a preproenzyme which then loses its N-terminal signalling peptide domain at the endoplasmic reticulum. This is followed by cleavage of the propeptide domain, which plays an important role in minimising deleterious premature activity by blocking active site access to potential substrates (Nissler et al., 2002). Indeed, use of recombinant CTSS propeptide has been investigated as a tactic to inhibit tumour advancement (Burden et al., 2008). CTSS is autocatalytically activated, whilst the presence of glycosaminoglycans (GAGs) has been shown to increase the rate of activation through ionic interaction with cationic propeptide residues (Vasiljeva et al., 2005). CTSS can be both activated within the lysosome, where the reduction in pH weakens the interaction between propeptide and active enzyme, and at extracellular neutral pH. Here, histidine deprotonation within the propeptide domain (which is particularly histidine rich in CTSS), has been suggested to weaken propeptide binding to the CTSS enzyme, facilitating increased interaction with GAGs (Nissler et al., 1999; Vasiljeva et al., 2005; Smyth et al., 2022). We hypothesise that our lead vNAR clones interact with the propeptide region of the proenzyme, stabilising it, and in turn, preventing autocatalysis. To the best of our understanding this is a novel approach towards CTSS inhibition and opens a range of exciting possibilities not just in cathepsin biology but in the application of the unique binding properties of vNARs for other targets.

Following this assessment of the mechanism behind our vNAR inhibition, we sought to prove the utility of our compound intracellularly. To achieve this, an intrabody approach was employed, with mammalian expression vectors encoding our lead clones designed and transfected into a panel of cell lines *in vitro* after which, successful expression was confirmed by Western blot. Assessment of CTSS levels post transfection revealed reduced levels of active CTSS, highlighting that our clones could not only be expressed intracellularly, but were sufficiently robust and appropriately trafficked to retain their functionality. vNARs were translated and folded correctly, facilitating target engagement, whilst the interaction of our vNAR clones with CTSS illustrates that our clones are appropriately trafficked to the target, validating the inclusion of the CTSS signal peptide motif in the expression vector.

To further illustrate the functional impact of our vNAR clones, we conducted an *in vitro* cell invasion assay. As stated, CTSS has been shown to play an important role in facilitating tumour cell invasion through proteolytic action upon the basement membrane (BM) and the ECM. This has been demonstrated previously in a RIP1-Tag2 murine pancreatic islet cancer model, wherein, knocking out CTSS led to a reduction in invasion, as a result of a diminution in E-cadherin cleavage (Gocheva et al., 2006). *In vitro*, invasion assays similar to that employed within this study, have been used to investigate the effects of CTSS on invasion of colorectal, breast and prostate cancer cell lines (Burden et al., 2009; Wilkinson et al., 2016). Here, we employed the malignant glioblastoma line U251. CTSS has been shown to play a role in the progression of glioblastoma with poorer outcomes associated with increased CTSS expression (Flannery et al., 2003; 2006). We observed that treatment with our vNAR clones attenuated the invasive nature of U251 cells, across an ECM mimetic matrix. CTSS has been shown to be secreted extracellularly as the proenzyme in a range of cell types including macrophages and tumour cells (Nissler et al., 1999; Sevenich et al., 2014; Wartenberg et al., 2019). Taken together these data suggest that in this assay, proCTSS is secreted extracellularly by the cells and is prevented from becoming activated by our lead vNAR clones.

Although inhibition of invasion was less pronounced than previously reported with small molecule approaches, the use of vNAR clones as demonstrated here may have other advantages (Wilkinson et al., 2016). Toxicology issues have been previously reported with small molecule cathepsin inhibitors, where lysosomal accumulation above therapeutically useful levels was noted (Falgueyret et al., 2005; Black and Percival, 2006). Whilst relatively resistant to degradation when compared to conventional biologics, vNARs are likely to be more prone to lysosomal break-down and in turn, provide less issues related to undesirable accumulation than small molecule compounds. Furthermore, the ability to utilise the vNAR constructs in an intrabody approach may prove an attractive treatment option, where small molecule inhibitors may struggle to successfully target lysosomal compartments.

Whilst the initial results stated here provide promise, further studies are required to fully elucidate the therapeutic potential of our vNAR clones. Firstly, the specificity and functionality of our clones will be assessed in a range of *in vivo* tumour models, through which pharmacokinetics, including accumulation, will be determined. These investigations will be conducted alongside assessment of drug delivery options including optimisation of our intracellular expression vectors and nanoparticle systems. Furthermore, to maximise the potential of our clones, binding affinity, as determined by SPR, will require enhancement. This could be achieved by reformatting the clones into multivalent constructs, which we have previously demonstrated can drastically improve binding affinity and efficacy, and through affinity maturation, with particular focus on the CDR1 binding domain (Zielonka et al., 2014; Ubah et al., 2017). Additionally, structural analysis could further elucidate the binding interaction and in turn, mechanism of inhibition, between vNAR clones and proCTSS.

In conclusion, we have demonstrated the potential application of vNARs for the inhibition of intracellular drug targets, here exemplified by CTSS. By preventing the activation of the CTSS proenzyme, a new inhibitory mechanism has also been revealed with prospective utility as both a therapeutic and tool compound for biological investigations. We propose that through the use of novel vNARs, a panel of previously undruggable proteins of interest can be targeted via a biologic approach.

## Data Availability

All data supporting this study is included within the main article and/or Supplementary Material accompanying this paper.
