# Supplementary figures and images for: Evaluation of variable new antigen receptors (vNARs) as a novel cathepsin S (CTSS) targeting strategy
Source: Front Pharmacol. 2023 Dec 5;14:1296567. doi: 10.3389/fphar.2023.1296567 (PMC10728302; doi:10.3389/fphar.2023.1296567)

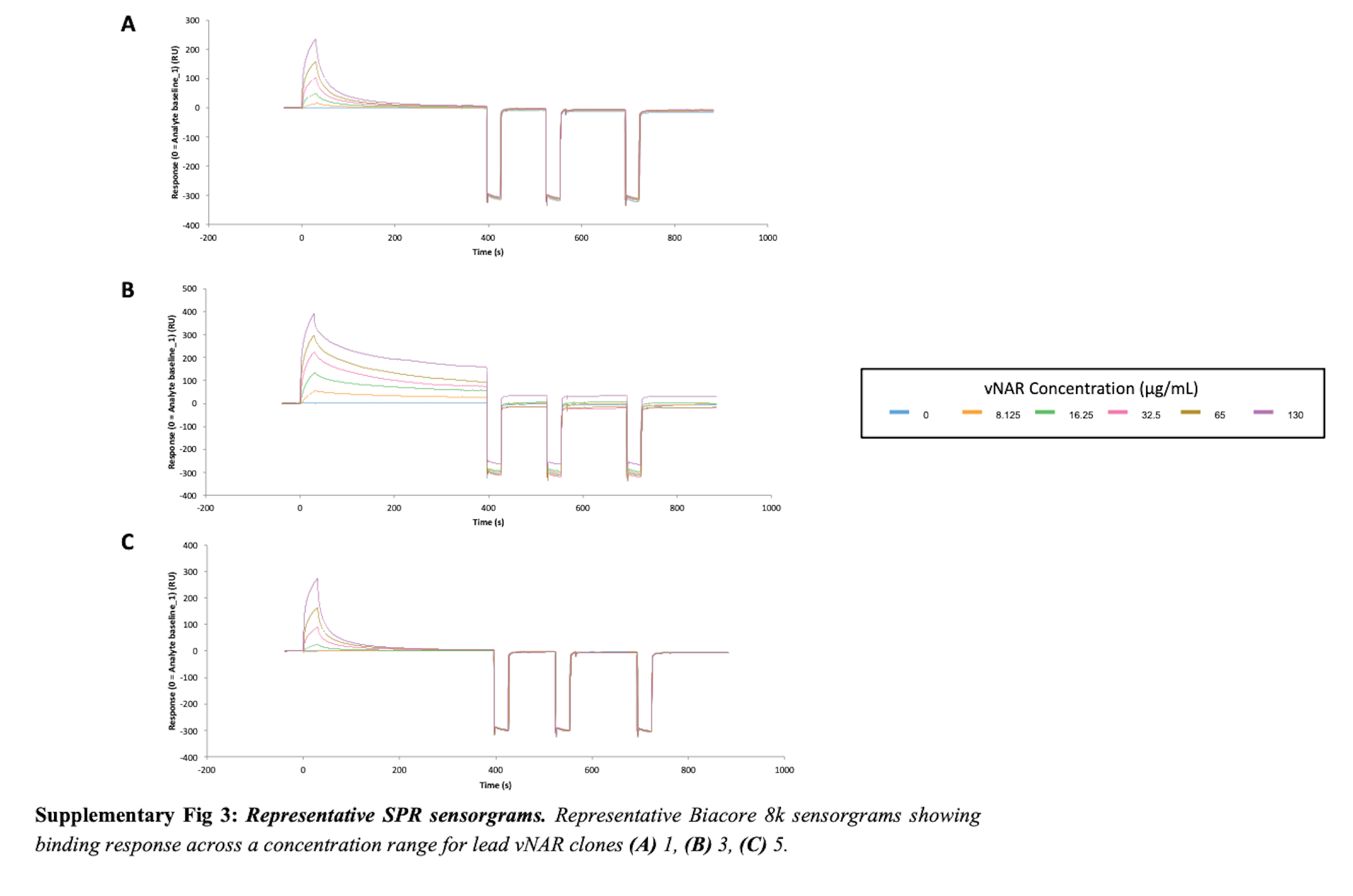

Supplement: Supplementary file 1 [file Image3.TIFF]

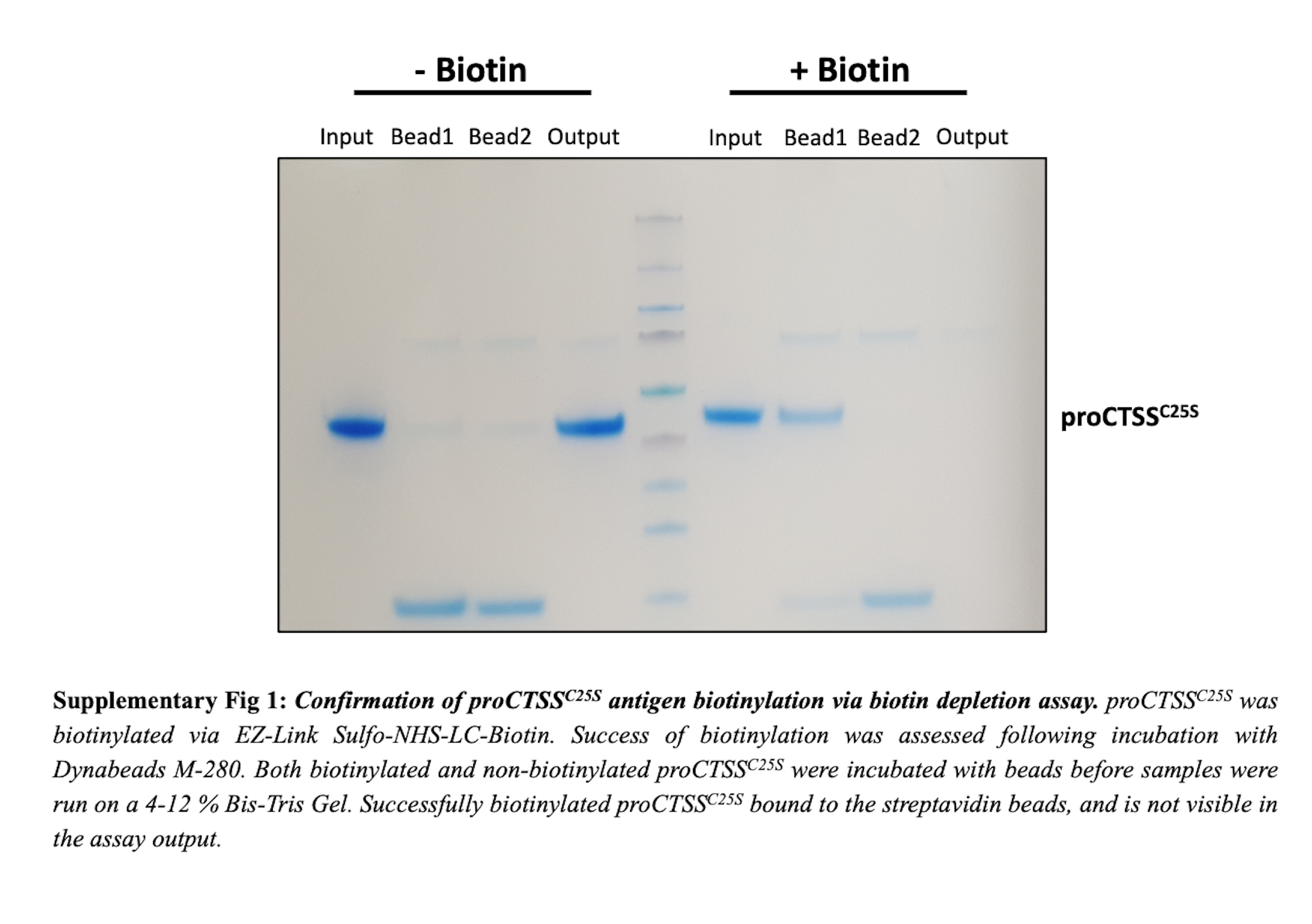

Supplement: Supplementary file 2 [file Image1.TIFF]

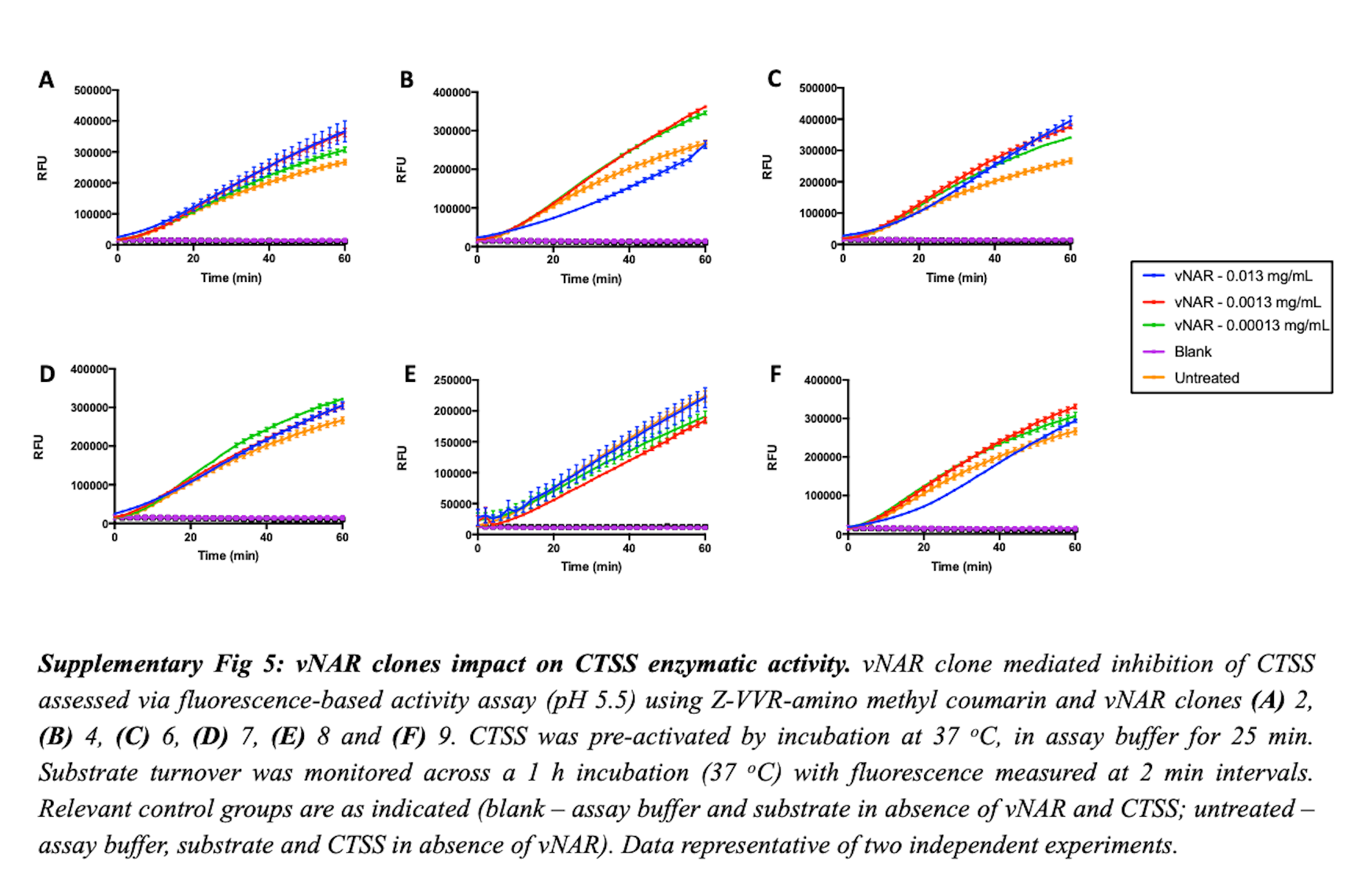

Supplement: Supplementary file 3 [file Image5.TIFF]

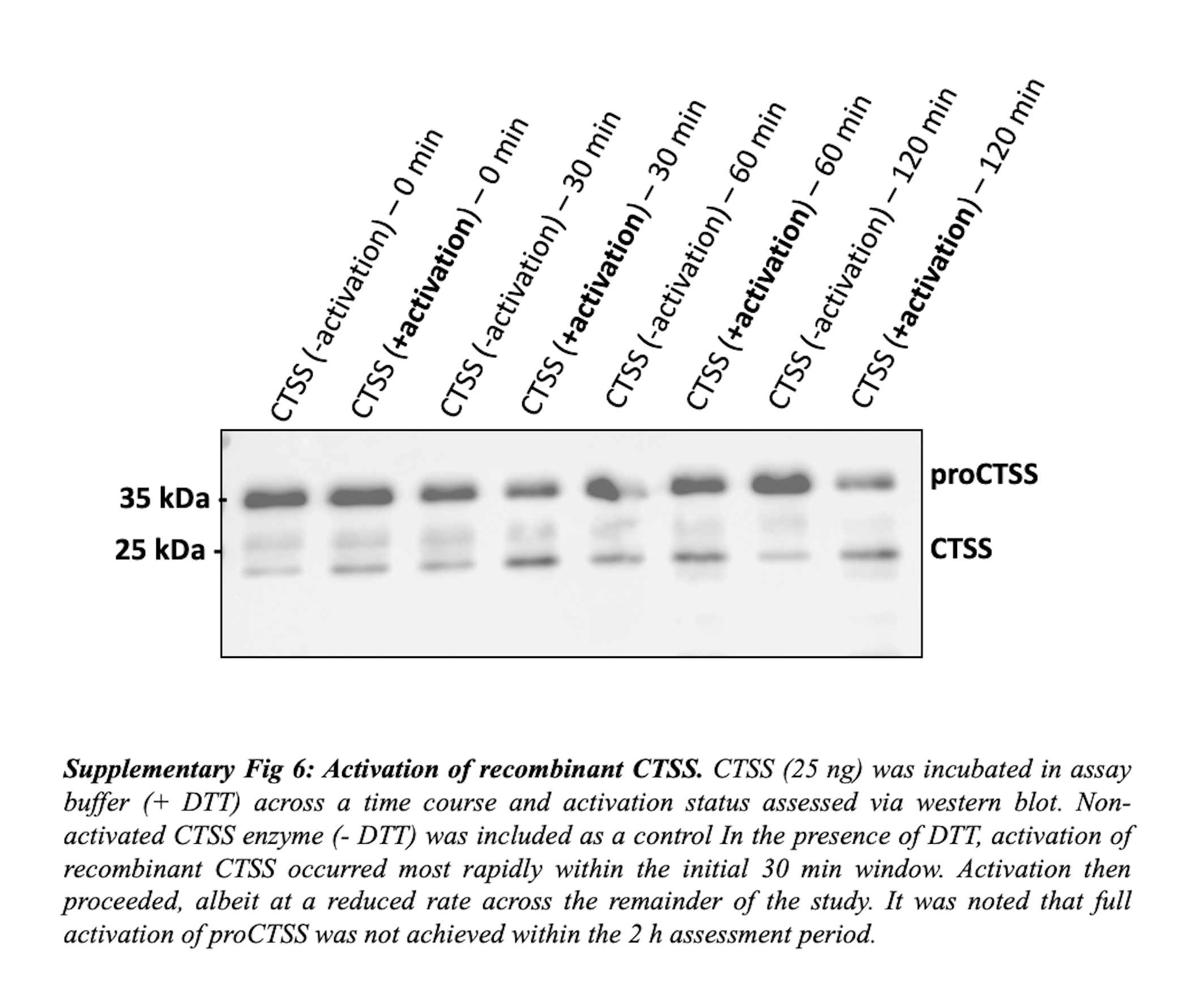

Supplement: Supplementary file 4 [file Image6.TIFF]

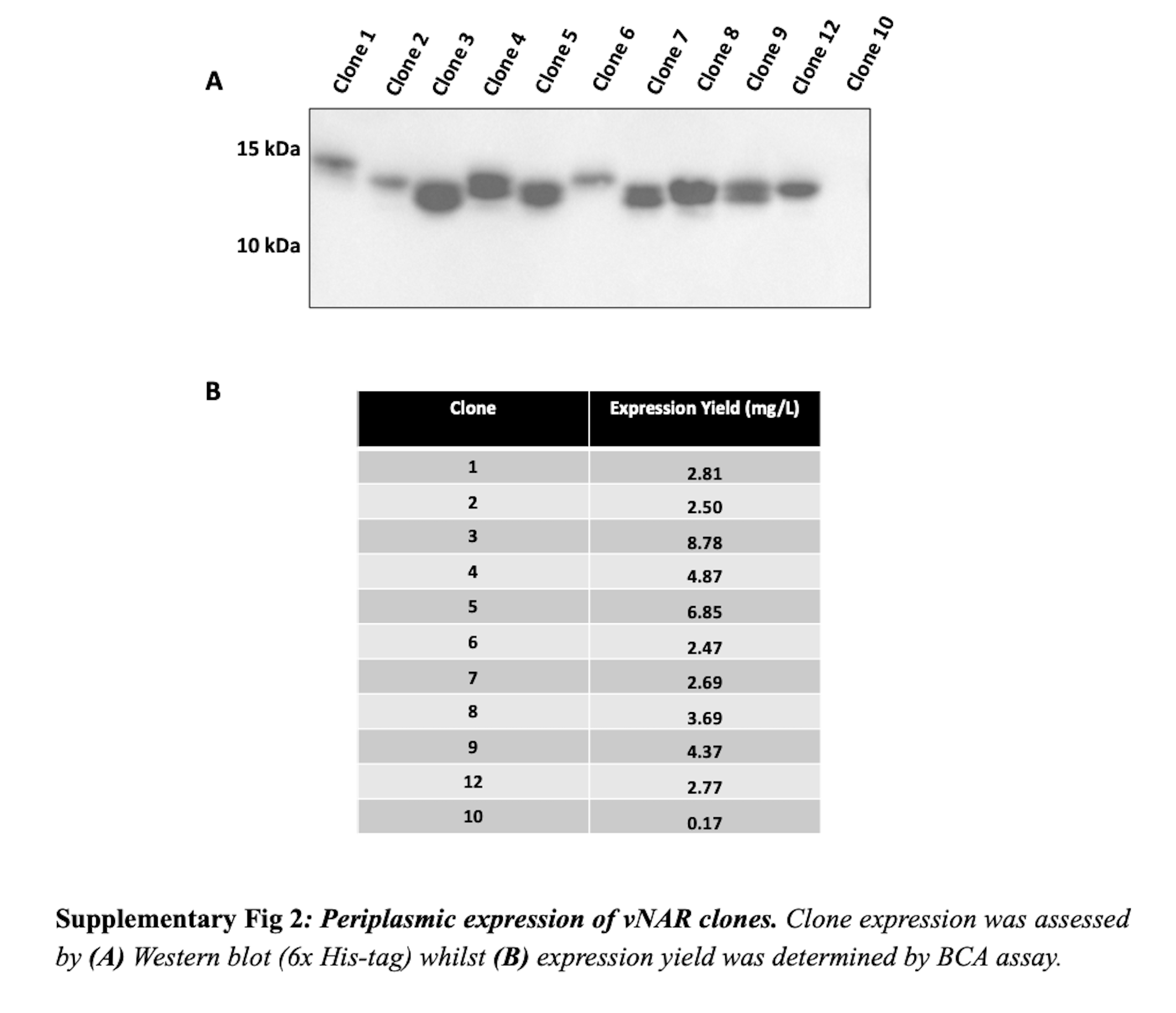

Supplement: Supplementary file 5 [file Image2.TIFF]

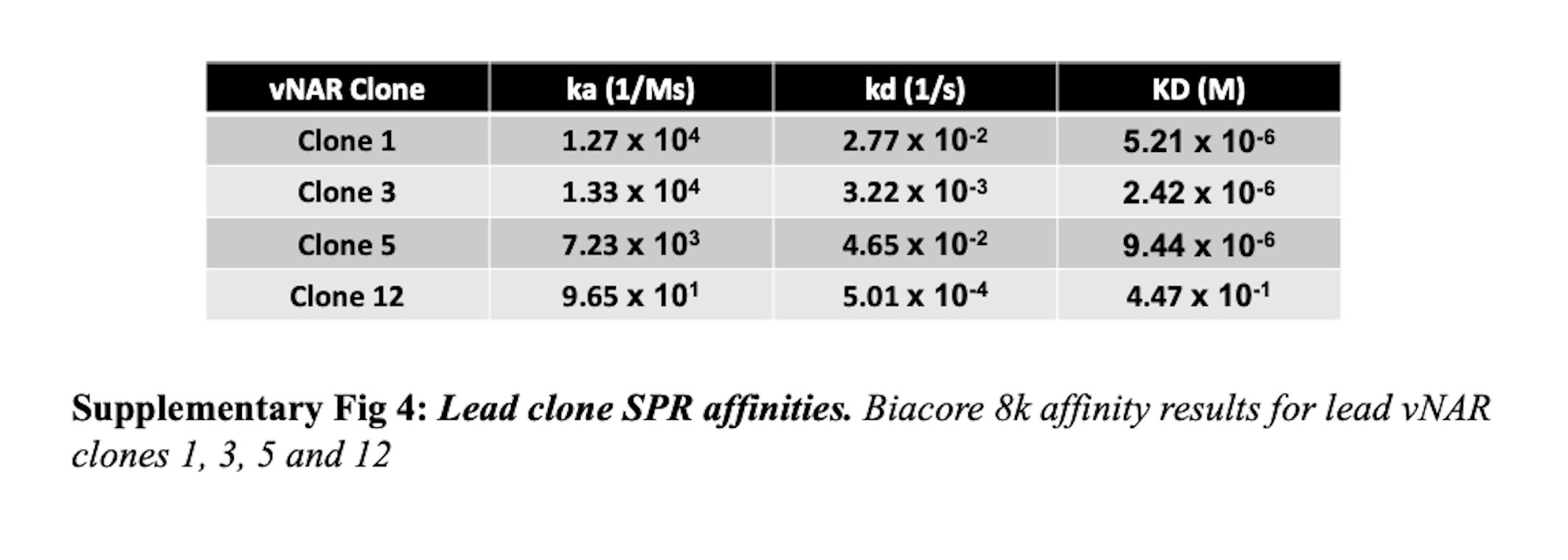

Supplement: Supplementary file 6 [file Image4.TIFF]

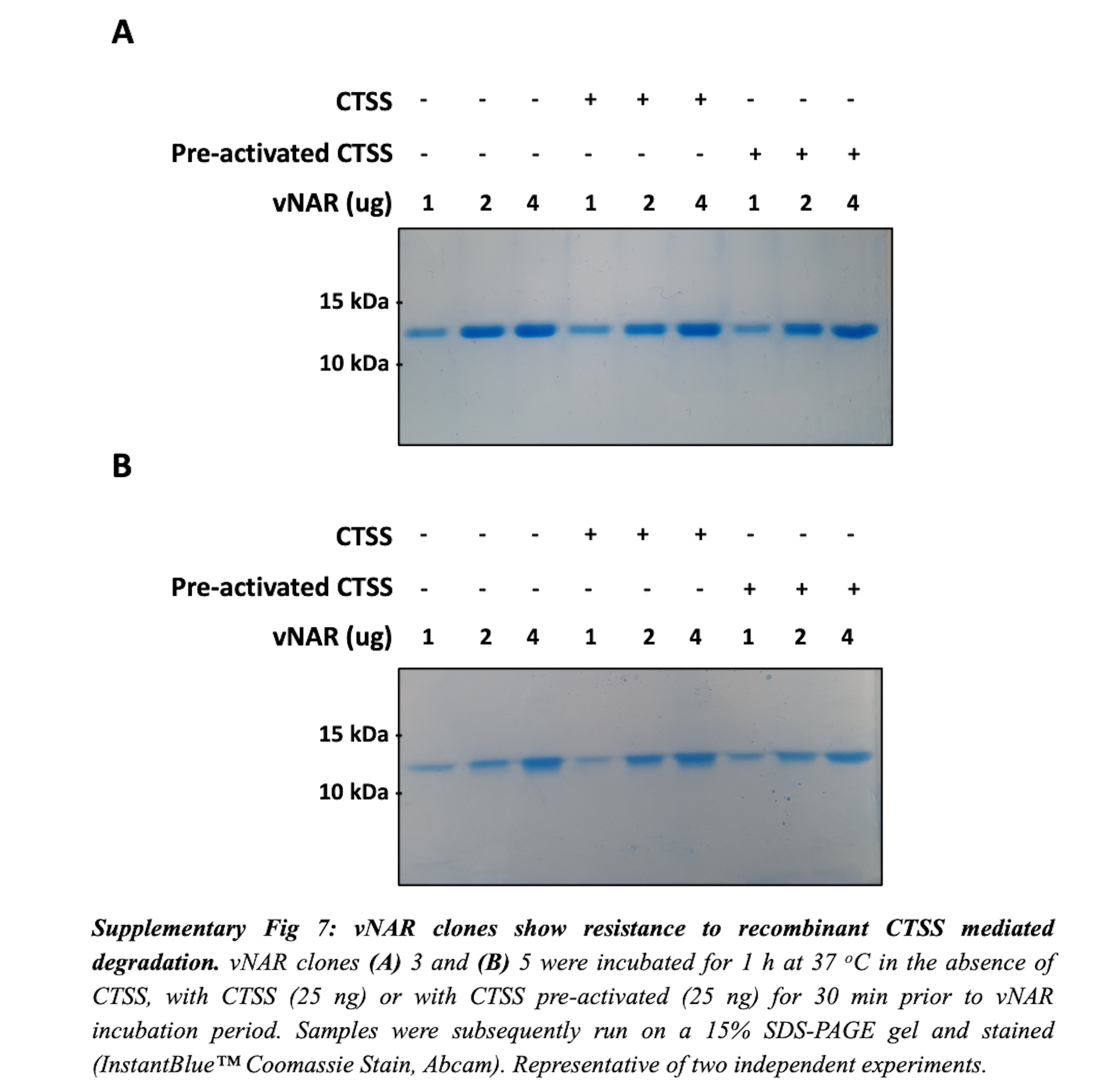

Supplement: Supplementary file 8 [file Image7.TIFF]
