# Supplementary material for: Evaluation of variable new antigen receptors (vNARs) as a novel cathepsin S (CTSS) targeting strategy
Source: Front Pharmacol. 2023 Dec 5;14:1296567. doi: 10.3389/fphar.2023.1296567 (PMC10728302; doi:10.3389/fphar.2023.1296567)
Supplement: Supplementary file 7 [file DataSheet1.docx]

**Supplementary Material - Evaluation of Variable New Antigen Receptors (vNARs) as a novel Cathepsin S (CTSS) targeting strategy**

**P. Smyth, L. Ferguson, J.F. Burrows, R.E. Burden, S.R. Tracey, Ú.M. Herron, M. Kovaleva, R. Williams, A.J. Porter, D.B. Longley, C.J. Barelle, C.J. Scott.**

**Supplementary Fig 1: *Confirmation of proCTSS^C25S^ antigen biotinylation via biotin depletion assay.*** *proCTSS^C25S^* *was biotinylated via EZ-Link Sulfo-NHS-LC-Biotin. Success of biotinylation was assessed following incubation with Dynabeads M-280. Both biotinylated and non-biotinylated proCTSS^C25S^ were incubated with beads before samples were run on a 4-12 % Bis-Tris Gel. Successfully biotinylated proCTSS^C25S^ bound to the streptavidin beads, and is not visible in the assay output.*

**Supplementary Fig 2*: Periplasmic expression of vNAR clones.*** *Clone expression was assessed by* ***(A)*** *Western blot (6x His-tag) whilst* ***(B)*** *expression yield was determined by BCA assay.*


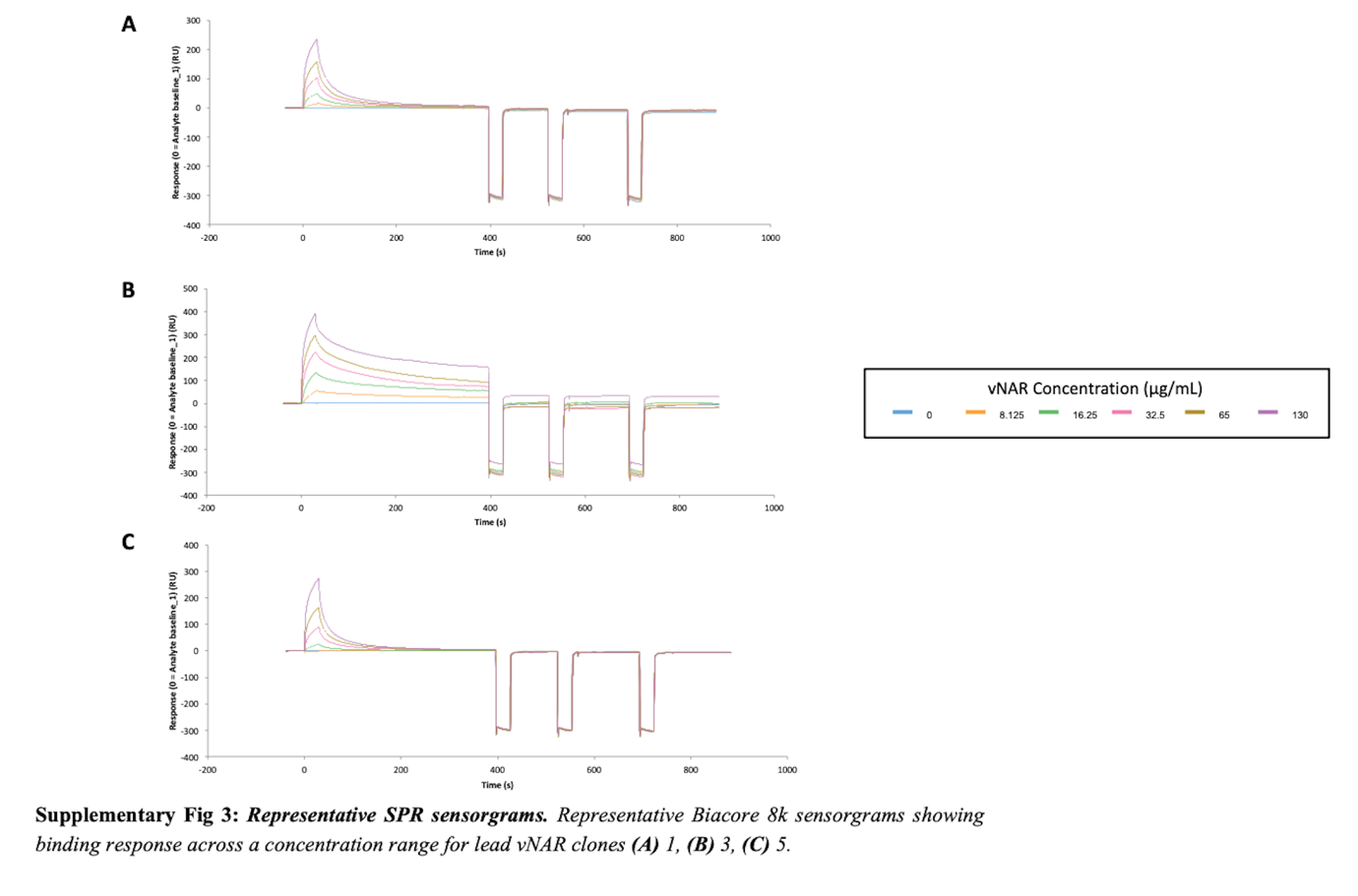


**Supplementary Fig 3: *Representative SPR sensorgrams.*** *Representative Biacore 8k sensorgrams showing binding response across a concentration range for lead vNAR clones* ***(A)*** *1,* ***(B)*** *3,* ***(C)*** *5.*

*
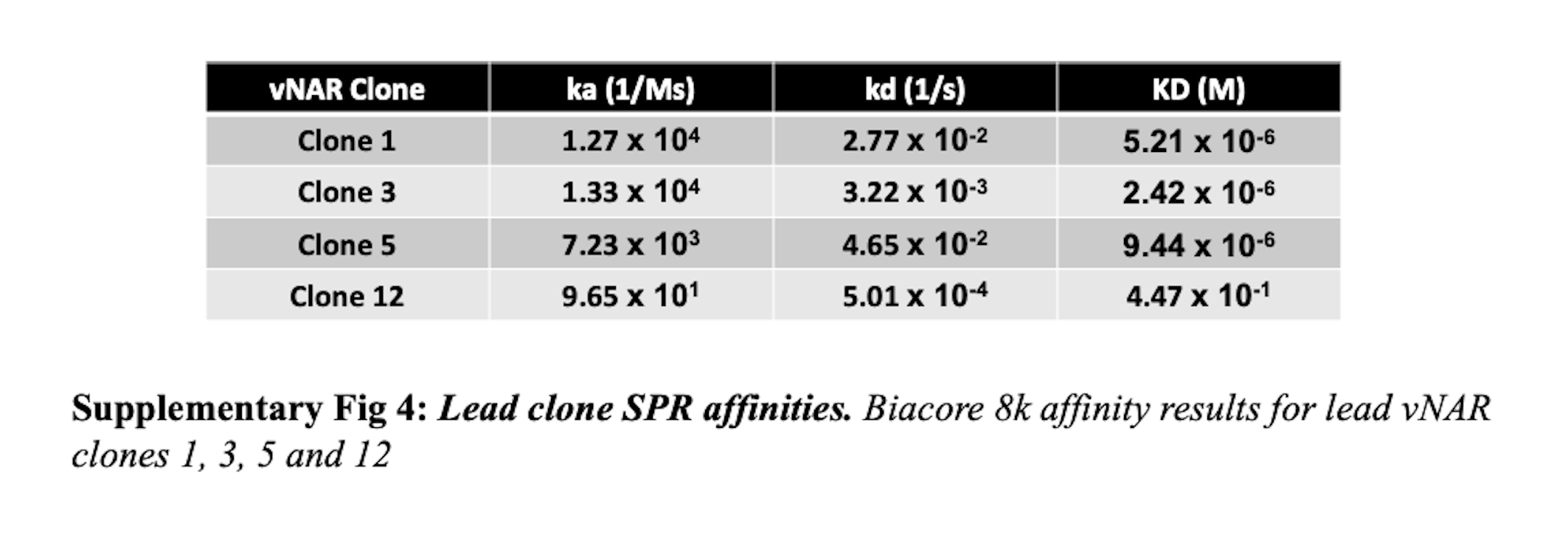
*

**Supplementary Fig 4: *Lead clone SPR affinities.*** *Biacore 8k affinity results for lead vNAR clones 1, 3, 5 and 12*

***Supplementary Fig 5:* *vNAR clone impact on CTSS enzymatic activity.*** *vNAR clone mediated inhibition of CTSS assessed via fluorescence-based activity assay (pH 5.5) using Z-VVR-amino methyl coumarin and vNAR clones* ***(A)*** *2,* ***(B)*** *4,* ***(C)*** *6,* ***(D)*** *7,* ***(E)*** *8 and* ***(F)*** *9. CTSS was pre-activated by incubation at 37 ^o^C, in assay buffer for 25 min. Substrate turnover was monitored across a 1 h incubation (37 ^o^C) with fluorescence measured at 2 min intervals. Relevant control groups are as indicated (blank – assay buffer and substrate in absence of vNAR and CTSS; untreated – assay buffer, substrate and CTSS in absence of vNAR). Data representative of two independent experiments.*

***Supplementary Fig 6: Activation of recombinant CTSS.*** *CTSS (25 ng) was incubated in assay buffer (+ DTT) across a time course and activation status assessed via western blot. Non-activated CTSS enzyme (- DTT) was included as a control. In the presence of DTT, activation of recombinant CTSS occurred most rapidly within the initial 30 min window. Activation then proceeded, albeit at a reduced rate across the remainder of the study. It was noted that full activation of proCTSS was not achieved within the 2 h assessment period.*

*.*

***Supplementary Fig 7: vNAR clones show resistance to recombinant CTSS mediated degradation.*** *vNAR clones* ***(A)*** *3 and* ***(B)*** *5 were incubated for 1 h at 37 ^o^C in the absence of CTSS, with CTSS (25 ng) or with CTSS pre-activated (25 ng) for 30 min prior to vNAR incubation period. Samples were subsequently run on a 15% SDS-PAGE gel and stained (InstantBlue™ Coomassie Stain, Abcam). Representative of two independent experiments.*
